# Adaptation and Evaluation of Traqq-Spain: An Ecological Momentary Dietary Assessment Smartphone App for the Spanish Adult Population

**DOI:** 10.1016/j.cdnut.2026.109397

**Published:** 2026-06-12

**Authors:** Mar Nafría, Hanne BT de Jong, Alice Chaplin, Dora Romaguera, Desiree A Lucassen

**Affiliations:** 1 Health Research Institute of the Balearic Islands (IdISBa), University Hospital Son Espases (HUSE), Balearic Islands, Spain; 2 Division of Human Nutrition and Health, Wageningen University & Research, Wageningen, The Netherlands; 3 Consorcio CIBER, M.P. Fisiopatología de la Obesidad y Nutrición (CIBEROBN), Instituto de Salud Carlos III, Madrid, Spain

**Keywords:** dietary assessment, ecologic momentary dietary assessment, Traqq, 2-h recall, food frequency questionnaire, technology evaluation

## Abstract

**Background:**

Diet is a key modifiable risk factor for chronic diseases, yet reliable assessment remains challenging. Ecological momentary dietary assessment applications, such as Traqq, enable real-time reporting, reducing recall errors and respondent burden compared with traditional methods. Originally developed in the Netherlands, Traqq requires cultural and linguistic adaptation before its use in other countries.

**Objectives:**

This study aimed to adapt Traqq to the Spanish context and evaluate its usability and accuracy for assessing habitual food group intake using random 2-h recalls (2hRs) compared with a validated food frequency questionnaire (FFQ) in Spanish adults.

**Methods:**

Traqq was adapted using a standardized approach, including the development of a country-specific food list linked to local food composition data (Base de Datos Española de Composición de Alimentos, US Department of Agriculture, local dishes), portion sizes (Centro de Enseñanza Superior de Nutrición y Dietética), interface translation, and pilot testing with refinement. The Spanish version was evaluated in 112 students at the University of the Balearic Islands. Participants completed repeated random 2hRs over 4 wk and an adapted FFQ. Agreement between methods was assessed using Wilcoxon signed-rank tests, Spearman correlations, quartile cross-classification, and Bland–Altman plots. Usability was evaluated using the System Usability Scale (SUS).

**Results:**

Seventy-nine participants were included in the analysis (mean age 24.9 ± 6.7 y; 73% female; 77% healthy body mass index; 67% not following a specific diet). Correlations between methods were good for most food groups (*r* = 0.52–0.65), except whole grains (*r* = 0.04). Mean intake of “Fruits and vegetables,” “Whole grains,” and “Fast and processed savory foods” was lower with 2hRs than FFQ. Traqq-Spain showed above-average usability (mean SUS = 78.9 ± 11.7).

**Conclusions:**

Traqq was successfully adapted for Spanish younger adults, demonstrating good agreement with a validated FFQ and high usability, supporting its use for real-time dietary assessment in this population.

## Introduction

Diet is one of the main modifiable risk factors for chronic diseases, yet its reliable assessment remains challenging. Dietary intake is usually assessed using self-reported methods, such as 24-h recalls (24hR), food frequency questionnaires (FFQ), and food records [1]. Although each method has its limitations, repeated 24hRs are often preferred over FFQs or food records for the assessment of dietary intake on an international level, because they allow standardized data collection across countries [2]. Still, 24hRs are highly prone to memory-related errors, which may lead to misreporting [[3], [4], [5]].

To overcome these limitations, ecological momentary dietary assessment (EMDA) is becoming increasingly accepted. EMDA, based on the principles of ecological momentary assessment, collects (near) real-time data on eating behaviors and contextual factors through smartphone applications (apps). It minimizes recall errors by prompting participants to record their food intake immediately or shortly after consumption, thereby offering an alternative to traditional dietary assessment methods [1,3,6]. Importantly, it offers shorter recall periods (e.g., 2-h, 4-h), reducing reliance on participants’ memory, shortening completion time, and decreasing participant burden [7].

Traqq is an EMDA smartphone app (iOS/Android) developed by Wageningen University (the Netherlands) for the Dutch adult population [8], designed to be tailored to different research needs. Unlike many other dietary assessment apps, Traqq is flexible in terms of reporting methods, supporting both food records as well as repeated short recalls [7]. Moreover, Traqq allows integrating additional food intake–related questions (e.g., contextual factors) [8]. Briefly, Traqq prompts users to report their food intake directly in the app. After opening it, a search screen opens, and consumed foods can be selected from an extensive food list based on the national food composition database [9]. After this, participants can be prompted to report consumed quantity, eating occasion, time of consumption, and any additional questions specified by the study settings.

To promote standardization across countries, it is preferable to adapt existing tools rather than develop new ones [10]. For cross-cultural applicability, integrating a country-specific food composition database is essential. Therefore, to enhance the usability of self-administered tools, a user-friendly, country-specific food list should be developed that is linked to the national food composition database [8,11]. Participants must be able to identify consumed foods easily, which requires a trade-off between comprehensiveness, clarity, and searchability (i.e., food descriptions must be clear, understandable, and easy to locate), particularly in an app [8].

Recently, researchers from the Health Research Institute of the Balearic Islands developed a screener to assess adherence to the 2018 World Cancer Research Fund (WCRF)/American Institute for Cancer Research (AICR) Cancer Prevention Recommendations [12]. To support the validation of this screener, an appropriate dietary assessment method is required as a reference. Therefore, we aimed to adapt Traqq to the Spanish context and evaluate its usability and accuracy for assessing habitual food group intake using random 2-h recalls (2hRs), compared with a validated FFQ, in a Spanish adult population.

## Methods

### Adaptation of Traqq for use in Spain

The adaptation process of Traqq to the Spanish context followed a standardized protocol based on the development of the original Dutch food list [8] and the adaptation of other dietary assessment tools for use in different countries [10,13]. The process included 4 steps: *1*) development of a country-specific food list linked to local food composition databases; *2*) development of country-specific portion size suggestions; *3*) translation of the app; and *4*) integration, pilot testing, and refinement. The adaptation process was conducted by a team of nutrition scientists, including experts on Traqq and experts on Spanish dietary habits.

The Spanish food list for Traqq was primarily based on the Base de Datos Española de Composición de Alimentos (BEDCA) v. 2.1 (BEDCA network) [14]. BEDCA is a public Spanish food composition database developed by the BEDCA network in 2005, following the standards set by the EuroFIR Network of Excellence on Food Composition Databank systems. It contains 992 food items and provides energy and 38 macro- and micronutrients for each item.

To make the BEDCA items suitable for use in Traqq, the adaptation process conducted included: *1*) removal of food items that cannot be consumed in their current form (e.g., raw pasta, coffee powder, raw fish); *2*) removal of infant foods and herbs and additives (without nutritional value); *3*) merging of food items with similar nutrient composition (<10% difference in nutrient values); *4*) removal of food items that are difficult to buy and/or rarely consumed; and *5*) replacement of technical food item descriptions to easily understandable names.

Furthermore, because the BEDCA database does not include novel Spanish foods, a total of 53 food items from the USDA’s FoodData Central database were added [15] (Supplemental Table 1). In addition, the team compiled a list of 12 of the most consumed dishes in Mallorca, Spain, which were not included in the used databases, and added these recipes to the database, together with their nutrient profile (Supplemental Table 2). The final Spanish food list consists of 831 food items.

Information on portion sizes and household measures for Traqq-Spain was obtained from the database of the Centro de Enseñanza Superior de Nutrición y Dietética (CESNID) [16], as well as from labeling information provided by the manufacturers. For a few missing foods, portion sizes were taken from the Dutch portion size database [17], which were checked by Spanish experts for suitability.

To allow use of Traqq in Spain, the app’s user interface required translation. All implemented text was translated by a native Spanish speaker and was checked for correctness. Finally, the newly developed food list with portion size suggestions was integrated into the Spanish version of Traqq and thoroughly checked by Spanish nutritionists. On the basis of this expert review, final refinements were made to Traqq-Spain.

### Evaluation of Traqq-Spain

#### Participants and study design

The evaluation of Traqq-Spain was embedded in a larger study being conducted for the validation of a novel diet and lifestyle screener for cancer prevention based on the WCRF/AICR 2018 Cancer Prevention Recommendations, in different study populations [12]. Traqq-Spain was evaluated in a convenience sample of 112 participants recruited at the University of the Balearic Islands between May 2023 and November 2023. Inclusion criteria were as follows: participants aged 18–45 y; enrolled at the University of the Balearic Islands or adjunct colleges; able to speak and read Spanish; and in possession of a smartphone with internet and the capability to use it.

At the start of the study, participants completed a brief sociodemographic questionnaire. Next, all participants were prompted at random days and times to report their food intake in Traqq during the 4-wk study period. At the end of the study period, participants were invited to complete a semiquantitative FFQ and an evaluation questionnaire. The study was approved by the Research Ethics Committee of the University of the Balearic Islands (No. 325CER23).

#### Traqq–random 2-h recalls

**FIGURE 1:**
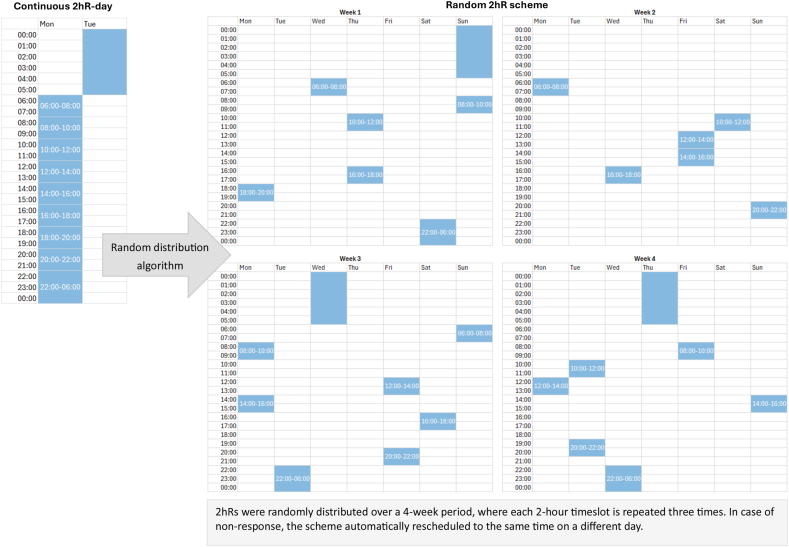
Example of a random 2hR scheme (right) based on a full day of 2hRs (left). 2hR, 2-h recall.

Habitual intake was measured by repeated random 2hRs over a 4-wk study period, using Traqq. Within this period, participants were randomly invited to report their food intake for the previous 2 h. An automated 2hR sampling scheme was created in Traqq for each participant. First, a 24-h template consisting of consecutive 2hRs was defined (on average 8 daytime 2hRs between 06:00 and 22:00), followed by an additional nighttime recall covering 22:00 to 06:00 and linked to the final evening 2hR. Subsequently, the individual time slots from this 24-h template were randomly distributed across the 4-wk study period. Each time slot was assessed 3 times, twice on weekdays and once on a weekend day (Figure 1). This approach was based on the traditional approach of using three 24hRs to assess habitual intake and has been extensively evaluated in Dutch settings [18].

To reduce participant burden, a maximum of 2 recalls per day was allowed. For all recalls, participants had a 1-h response deadline, except for the nighttime recall, which had a 2-h response deadline. All invitations were scheduled between 06:00 and 22:00, with the exception of the nighttime food intake recall. In case of nonresponse, the 2hR closed, and a new invitation was automatically rescheduled for the same timeslot on a different day. After 4 wk, the scheme ended, and responses were checked for completeness, with >21 completed 2hRs considered equivalent to 3 valid days, in line with previous evaluation of the random 2hR approach in the Dutch context [18].

Data from the 2hRs were entered in the computation module of Compl-eat (Wageningen University, the Netherlands), which also includes the previously described expansion of the BEDCA [19]. Dietary intake data were thoroughly checked by trained dieticians, according to a standardized protocol. The review focused on completeness and identification of implausible amounts. Errors were corrected according to a standardized approach, using standard portion sizes and recipes (e.g., 150 cups of tea was corrected to 1 cup of 150 g). Participants were not contacted in case of discrepancies.

Habitual intake was calculated for each participant by adding up reported intake of all the 2hRs and dividing them by 3, because each time point was assessed 3 times. Underreporters and overreporters were identified and excluded using the Willet cut-offs (i.e., males with energy intakes <800 kcal or >4200 kcal; females <500 kcal or >3500 kcal) [20]. Reported foods and drinks were grouped into the following categories according to the WCRF/AICR recommendations: “Fruits and vegetables,” “Whole grains,” “Legumes,” “Fast and processed foods (savory),” “Fast and processed foods (Sweet),” “Red meat,” “Processed meat,” “Sugar-sweetened drinks,” and “Alcoholic drinks” (Supplemental Table 3).

#### Food frequency questionnaire

An adaptation of a validated semiquantitative FFQ for the Spanish population [[21], [22], [23]] was used as a reference method. The adaptation consisted of including only food groups relevant to cancer prevention, based on the WCRF/AICR recommendations [i.e., “Fruits and vegetables,” “Whole grains,” “Legumes,” “Fast and processed foods (savory/sweet),” “Red meat,” “Processed meat,” “Sugar-sweetened drinks,” and “Alcoholic drinks”], and updating the list of food items to include novel foods in the Spanish food system. The FFQ included a total of 91 food items and 9 categories of frequency of consumption, ranging from “never or almost never” to “7 or more times per day.” Portion sizes were estimated using standard portions and commonly used household measures. The FFQ was interviewer-administered by a trained nutritionist, who completed the questionnaire based on the participant’s responses. FFQ data were calculated by multiplying portion size by frequency of consumption to obtain average daily intake (in grams) of each food item. Trained dieticians conducted multiple quality checks to safeguard the quality of the data.

#### Usability assessment

At the end of the study period, usability of Traqq-Spain was assessed using the System Usability Scale (SUS), which is a commonly used method to assess usability of digital tools, including dietary assessment apps [24,25]. The SUS contains 10 statements related to perceived usability, and for each statement, respondents rate their level of agreement on a Likert scale ranging from 1 (strongly disagree) to 5 (strongly agree) [26]. The SUS was administered anonymously, preventing in-depth analyses of usability across participant characteristics such as age or gender.

#### Other variables

General participant characteristics were collected via a sociodemographic questionnaire at the start of the study, including age, sex, educational level, living situation, country of origin, cooking habits, and dietary habits. Height and weight were measured by a trained dietician. Height was measured without shoes using a stadiometer (ADE MZ10017, ADE), and weight was measured by bioimpedance, without shoes, heavy clothing, and empty pockets (Tanita BC-148, Tanita). BMI was calculated as weight (kg) divided by height squared (m^2^), according to the standard formula.

#### Statistical analyses

Population demographics are presented as means with SDs and frequencies (*n*) with percentages (%). The SUS score was calculated with a predefined formula (range 0–100). A SUS score of >68/100 indicates an above-average usability, and a score of >80/100 indicates an excellent usability [26].

To evaluate the agreement between the random 2hRs and the FFQ, for intake of prespecified food groups, multiple statistical tests were used [27]. The absolute mean intake difference between the 2hR and the FFQ was calculated and evaluated using Wilcoxon signed-rank tests. Spearman correlation coefficients were calculated to assess the strength and direction of the association between the mean intake assessed with 2hRs and FFQ. Correlation coefficients of <0.20 were classified as poor, 0.20–0.49 as acceptable, and ≥0.50 as good [27]. The ranking ability of the 2hRs was evaluated by dividing the intake of foods as assessed by both the 2hRs and FFQ into quartiles. We then examined whether persons were ranked in the same, same or adjacent, or extreme quartile. Classification of ≥50% of the participants in the same quartile, ≥75% in the same or adjacent quartile, and <10% in the extreme quartile was considered a good outcome [27]. Agreement between methods was additionally assessed using weighted kappa statistics based on quartile classification, where values of 0.21–0.40 indicate fair agreement, 0.41–0.60 indicate moderate agreement, 0.61–0.80 substantial agreement, and >0.80 almost perfect agreement [27,28]. Presence, direction, and the extent of bias at the group level for the different food groups were visualized in Bland–Altman plots, where the difference between the 2hRs and FFQ was plotted against the mean of the 2 methods [27,29]. Sensitivity analyses were performed, including participants with fewer than 21 completed 2hRs and those identified as underreporters or overreporters. These analyses yielded similar results to the main analyses, indicating that exclusion of these participants did not materially affect the findings. All analyses were performed using IBM SPSS Statistics version 28.0.1.1 and Stata version 17.0 (StataCorp LLC).

## Results

### Participant characteristics

A total of 112 participants were initially included; 3 were unable to download or set up the app (i.e., limited memory space and outdated software), 23 did not complete ≥21 single 2hRs (Supplemental Figure 1), and 7 were excluded due to misreporting (6 overreporters and 1 underreporter), resulting in a final sample of 79 participants. Their mean ± SD age was 24.9 ± 6.7 y, 73% were female, and 77% had a healthy BMI. Overall, 52% lived with their parents or other relatives, 23% with a partner, and 25% with others or alone. More than half (57%) cooked their own meals regularly. Most participants (67%) did not follow a specific diet, whereas 10% practiced intermittent fasting, 6% were vegetarian, and 3% were vegan (Table 1).

**TABLE 1:** General characteristics of the participants included in the validation study.

	Total (79)	Males (21)	Females (58)
Mean age, y (SD)	24.9 (6.7)	23.6 (4.1)	25.4 (7.4)
Mean BMI, kg/m^2^ (SD)	22.8 (3.6)	24.2 (3.7)	22.3 (3.4)
BMI category, *n* (%)			
≤18.5 kg/m^2^	0 (0)	0 (0)	0 (0)
18.5–24.9 kg/m^2^	61 (77)	14 (67)	47 (81)
25.0–29.9 kg/m^2^	14 (18)	6 (28)	8 (14)
≥30 kg/m^2^	4 (5)	1 (5)	3 (5)
Level of study, *n* (%)			
Undergraduate	46 (58)	13 (62)	33 (57)
Postgraduate	33 (42)	8 (38)	25 (43)
Living situation, *n* ( %)			
With parents or other relatives	41 (52)	12 (56)	29 (50)
With partner	18 (23)	2 (10)	16 (28)
Roommates	12 (15)	5 (24)	7 (12)
Alone	6 (8)	1 (5)	5 (8)
Other	2 (2)	1 (5)	1 (2)
Country of origin, *n* (%)			
Spain	63 (80)	14 (67)	49 (84)
Other country	16 (20)	7 (33)	9 (16)
Cooking, *n* (%)			
Usually cooks	45 (57)	12 (57)	33 (57)
Usually does not cook	34 (43)	9 (43)	25 (43)
Dietary habits, *n* (%)			
Not following a specific diet	54 (69)	15 (71)	39 (66)
Intermittent fasting	8 (10)	3 (14)	5 (8)
Vegetarian	5 (6)	1 (5)	4 (6)
Flexitarian	2 (3)	1 (5)	1 (2)
Vegan	2 (3)	0 (0)	2 (4)
Diet without lactose	2 (3)	0 (0)	2 (4)
Diet for weight loss	1 (1)	1 (5)	0 (0)
Diet without gluten	1 (1)	0 (0)	1 (2)
Diet without nuts	1 (1)	0 (0)	1 (2)
Diet without alcohol	1 (1)	0 (0)	1 (2)
Halal	1 (1)	0 (0)	1 (2)
Fodmap diet	1 (1)	0 (0)	1 (2)

### Agreement of food groups reported with random 2hRs compared with FFQ

**FIGURE 2:**
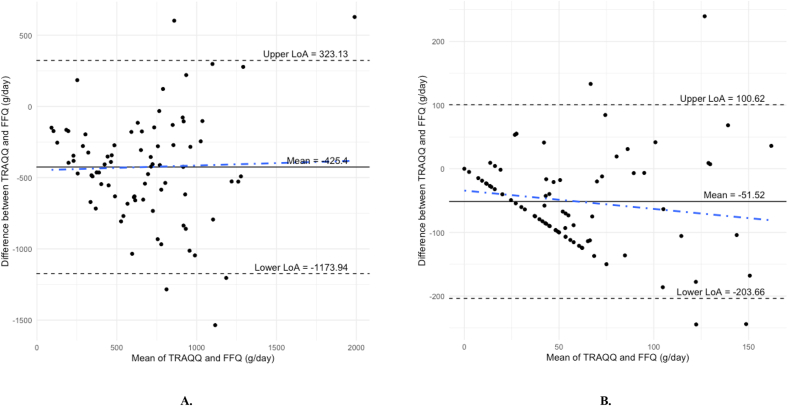
Bland–Altman plots for the differences in intake estimated with the 2hRs and the FFQ for the food groups “Fruit and vegetables” (A) and “Whole grains” (B), plotted against the mean of both methods (g/d). Mean difference (solid line), 95% limits of agreement (1.96 × SD of mean difference; dashed line), and linear regression line (blue dashed line) are included. 2hR, 2-h recall; FFQ, food frequency questionnaire; LoA, limits of agreement.

Mean reported consumption (g/d) of “Fruit and vegetables,” “Whole grains,” and “Fast and processed foods (savory)” was significantly higher with the FFQ compared with 2hRs (Table 2). No significant differences were found between other food groups when comparing the 2 methods. Furthermore, correlation between data obtained from the 2hRs and FFQ was good for “Fruit and vegetables” [*r* = 0.60; 95% confidence interval (CI): 0.42, 0.74], “Fast and processed foods (total)” (*r* = 0.52; 95% CI: 0.32, 0.67), “Fast and processed foods (sweet)” (*r* = 0.61; 95% CI: 0.43, 0.74), and “Alcoholic drinks” (*r* = 0.65; 95% CI: 0.49, 0.77). “Legumes,” “Red meat,” “Processed meat,” and “Sugar-sweetened drinks” showed acceptable correlations (*r* = 0.25–0.46), whereas “Whole grains” presented the lowest correlation (*r* = 0.04; 95% CI: –0.19, 0.25), which was in accordance with a higher misclassification in the extreme quartile (14%) and a poor agreement based on weighted kappa (0.13). “Legumes” also showed a relatively high misclassification (10%), whereas the rest of the food groups ranged between 0% and 6%. The Bland–Altman plots supported these findings. For “Fruit and vegetables” and “Whole grains,” the plots showed systematically lower reported intakes with the 2hRs than with the FFQ (Figure 2). For all food groups, differences between the 2hRs and the FFQ tended to increase with increasing intake (Supplementary Figure 2).

**TABLE 2:** Mean intake of food groups (g/d) assessed by random 2hRs and FFQ with corresponding Wilcoxon signed-rank tests, Spearman correlation coefficients between the 2hRs and FFQ, and corresponding cross-classification (*n* = 79).

	2hRs	FFQ	*P*^1^	Correlation coefficient^2^ (95% CI)	Cross-classification by quartiles			Weighted kappa^3^ (κ)
	Mean ± SD	Mean ± SD			Same (%)	Same + adjacent (%)	Extreme (%)	
Fruit and vegetables	463 ± 394	888 ± 385	<0.001	0.60 (0.42, 0.74)	48	86	3	0.54
Whole grains	34 ± 51	86 ± 59	<0.001	0.04 (–0.19, 0.25)	27	56	14	0.13
Legumes	74 ± 78	74 ± 72	0.79	0.25 (0.03, 0.45)	33	72	10	0.23
Fast and processed foods (total)	146 ±110	145 ± 93	0.69	0.52 (0.32, 0.67)	49	82	3	0.49
Fast and processed foods (savory)	75 ± 77	89 ± 65	0.03	0.27 (0.05, 0.47)	30	65	6	0.16
Fast and processed foods (sweet)	71 ± 66	56 ± 42	0.07	0.61 (0.43, 0.74)	41	90	3	0.59
Red meat	49 ± 69	39 ± 39	0.36	0.46 (0.26, 0.63)	37	76	6	0.37
Processed meat	29 ± 48	27 ± 25	0.67	0.42 (0.21, 0.59)	39	76	4	0.39
Sugar-sweetened drinks	81 ± 127	56 ± 71	0.20	0.45 (0.25, 0.62)	30	84	4	0.43
Alcoholic drinks	82 ± 163	71 ± 117	0.94	0.65 (0.49, 0.77)	47	91	0	0.57

Abbreviations: 2hR, 2-h recall; CI, confidence interval; FFQ, food frequency questionnaire.

1 Wilcoxon signed-rank test comparing mean intakes assessed with 2hRs and FFQ.

2 Spearman correlation between the mean of the 2hRs and FFQ.

3 Weighted kappa was calculated to assess agreement between methods based on cross-classification into quartiles.

### Usability of the Spanish version of Traqq

**FIGURE 3:**
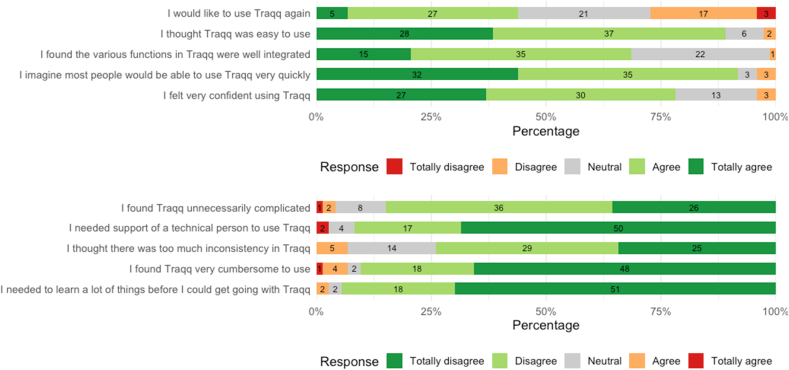
Distribution of the responses to each SUS item after using Traqq-Spain. SUS, System Usability Scale.

In total, 73 of the original 112 participants completed the SUS. The mean (SD) SUS score was 79 (12) of 100, indicating above-average usability. The response to each SUS item is presented in Figure 3, where the positive items (odd-numbered statements) and the negative items (even-numbered statements) have been grouped. Participants were generally positive about the app, with 89% (*n* = 65) finding Traqq-Spain easy to use and 95% (*n* = 69) mastering it quickly.

## Discussion

This study describes the adaptation and evaluation of Traqq for use in a Spanish adult population. First, a country-specific food list with portion size examples was developed and integrated into Traqq. Data obtained with the app-based 2hRs showed acceptable-to-good correlations with a validated semiquantitative FFQ across all food groups, except for “Whole grains.” Furthermore, an anonymous usability evaluation indicated that Traqq-Spain is an easy-to-use tool.

The comparisons between food group intakes estimated by the 2hRs, via Traqq-Spain, and the FFQ data showed consistent results across most categories. However, a large difference was found for “Fruit and vegetables,” with a relatively high intake reported via the FFQ (888 g/d compared with 463 g/d in Traqq). This overestimation in the FFQ can be attributed to social desirability bias when completing the FFQ. In addition, its structure, which allows multiple selections per item, may lead participants to indicate higher consumption of foods they consume only occasionally, thereby inflating reported intakes. Previous research supports these findings, as a meta-analysis concluded that FFQs are valid for assessing overall dietary patterns in epidemiological studies, although they tend to overestimate intake compared with other reference methods [30]. Moreover, previous data from a representative Spanish population aged 18–79 y reported average intakes of 158 ± 175 g/d for fruits and 178 ± 113 g/d for vegetables [31], suggesting that the FFQ values are considerably higher than expected. It is also important to note that participation in this study was voluntary, and individuals genuinely interested in healthy eating may have been more likely to take part, potentially introducing a bias toward healthier dietary patterns compared with the general population.

A large difference was also observed for “Whole grains,” with mean intakes of 86 ± 59 g/d reported in the FFQ compared with 34 ± 51 g/d in the 2hRs. To put this into context, a study in an older Spanish population estimated that individuals in the third quintile consumed on average 17 ± 32 g/d, whereas those in the highest quintile reached 89 ± 93 g/d [32]. These figures suggest a pattern similar to that observed for fruits and vegetables: the FFQ may overestimate habitual intake relative to short-term recalls. However, the whole grains group may be more prone to day-to-day variation than fruits and vegetables, as it comprises a wider variety of foods (e.g., wholegrain breads, wholegrain breakfast cereals, brown rice, and wholegrain pasta) that are not necessarily consumed daily and may therefore be more easily missed by the random 2hRs. Still, both methods fall within plausible ranges observed in population data. Overall, the remaining food groups showed relatively good agreement between methods, indicating that, despite specific discrepancies in “Fruits and vegetables” and “Whole grains,” the Traqq-Spain tool provides a comparable estimation as other validated assessments of habitual dietary intake.

Developing an appropriate food list linked to a reliable local food composition database is crucial not only to ensure that users can find consumed foods, but also to maintain the quality of the data output [8,10,33,34]. However, the available Spanish databases (i.e., BEDCA, CESNID) are over 20 y old. As consumption patterns have changed over time, clear trends have emerged, including higher meat intake, greater consumption of ready-made foods, and increased reliance on takeaway meals [35]. Therefore, it is likely that these databases are incomplete. We attempted to supplement missing foods from a more recent database (i.e., USDA version 2023); however, this database was originally developed for the United States. As food formulations differ between countries, including these foods may have affected the quality of the underlying database. These issues underscore the need for country-specific, reliable, and up-to-date food composition databases [36,37]. Nevertheless, it is unlikely that this limitation affected our outcomes, as both methods mostly relied on the same databases.

Usability was assessed using the SUS, an easy-to-use questionnaire to quickly assess usability of digital tools [26]. The SUS has been used in many studies to assess the usability of digital dietary assessment tools. The mean SUS score for Traqq-Spain was similar to the mean SUS score for the original, Dutch version of Traqq (79/100 compared with 72/100, respectively) [38]. The slightly higher score in Traqq-Spain is potentially due to the fact that several updates have been conducted because the Dutch validation to, among others, increase usability. Still, this SUS score is relatively similar to what has been found for other dietary assessment tools that were adapted for use in different countries [10,13,39].

The main strength of this study was the comprehensive approach used to adapt Traqq to the Spanish context, which involved both experts on Traqq and local nutrition scientists. However, several limitations should be noted. First, the study population consisted of university students from Mallorca, which may limit the generalizability of the findings to younger adults, particularly young females. For instance, high SUS scores may reflect a high level of digital literacy and familiarity with apps and digital tools. Nevertheless, this is likely representative of a large part of the population due to the increasing use of technology. Still, additional evaluation is recommended before applying the app in other Spanish populations, such as the elderly. Furthermore, Traqq-Spain was developed for a validation study of a newly developed screener on adherence to the WCRF/AICR recommendations. Consequently, we only present results for specific food groups, whereas Traqq can also be used to assess intake of energy, macronutrients, and micronutrients, as well as other food groups included in the current Spanish food database (BEDCA). For broader use, further evaluation is warranted.

To conclude, Traqq was successfully adapted for use in a Spanish adult population. To the best of our knowledge, this represents the 1st EMDA app developed for use in Spain. In this validation study, the app-based 2hRs demonstrated generally good agreement with the FFQ across most food groups. Usability of Traqq-Spain was rated as above-average, consistent with the original Dutch version. Nevertheless, a more extensive evaluation is warranted before applying Traqq-Spain in other Spanish populations.

## Author contributions

The authors’ responsibilities were as follows – MN, HBTdJ, AC, DR, DAL: designed research; MN, AC, DR: conducted research; MN, HBTdJ: analyzed data; MN, HBTdJ, AC, DAL: wrote the manuscript; DAL: primary responsible for the final content; and all authors: read and approved the final manuscript.

## Data availability

Data described in the manuscript, code book, and analytic code will be made available on request pending.

## Declaration of generative AI and AI-assisted technologies in the writing process

During the preparation of this work, the authors used ChatGPT in order to assist with grammar checking. After using this tool/service, the authors reviewed and edited the content as needed and take full responsibility for the content of the publication.

## Funding

This work was supported by the Health Research Institute of the Balearic Islands (IdISBa, SYN21/05). MN holds a predoctoral contract from the Health Research Institute from the Balearic Islands funded by the Sustainable Tourism Tax Fund (ITS) of the Government of the Balearic Islands (ITS2023-057).

## Conflict of interest

The authors report no conflicts of interest.
