# Supplementary material for: Adaptation and Evaluation of Traqq-Spain: An Ecological Momentary Dietary Assessment Smartphone App for the Spanish Adult Population
Source: Curr Dev Nutr. 2026 Jun 12;10(7):109397. doi: 10.1016/j.cdnut.2026.109397 (PMC13342963; doi:10.1016/j.cdnut.2026.109397)

Supplemental materials to

Nafria et al. Adaptation and evaluation of Traqq-Spain: An ecological momentary dietary assessment smartphone app for the Spanish adult population.

Supplemental Table 1. Food items added to the Spanish Traqq food list and its underlying food composition database (based on BEDCA) from the USDA's FoodData Central database.

Category	Spanish name	English name	Database ID
Beverages (non-dairy)	Agua, de sabores	Water, flavoured	14605
	Café, preparado, azucarado	Coffee, store-bought, sweetened	14179
	Té, preparado, azucarado	Tea, store-bought, sweetened	14206
Fruit (products)	Membrillo	Quince paste	817944010534
	Arándano, deshidratado	Cranberries, dried	62109100
Grain (products)	Pasta de maíz, hervida	Corn pasta	20092
	Pasta de lentejas, hervida	Lentil pasta	655635970039
	Biscote, integral	Melba toast, wholegrain	18221
	Picos	Breadsticks	51183990
	Picos, integrales	Breadsticks, wholegrain	51306000
	Cuscús integral, cocido	Couscous, wholegrain, cooked	3997805497
	Pan, sin gluten	Bread, gluten-free	51808000
	Tallarines de arroz, hervidos	Rice noodles, cooked	56117090
	Tortitas de arroz	Rice crackers	25071
	Tortilla de maíz, tipo taco	Taco shell, corn	52215300
	Tortilla de maíz, tipo fajita	Fajita tortilla, corn	52215100
	Tortilla de trigo, tipo fajita	Fajita tortilla, wheat	1903327
	Tortilla de trigo integral, tipo fajita	Fajita tortilla, wholegrain	52215260
	Raviolis de queso, hervidos	Pasta, filled with cheese, boiled	58131510
Meat (products)	Hamburguesa	Hamburger, cooked	21107
	Nuggets, de pollo	Nuggets, chicken	22975
Miscellaneous foods	Tempeh, cocinado	Tempeh, cooked	16174
	Fajita, de pollo	Fajita, chicken	58105000
	Fajita, de verduras	Fajita, vegetable	58105075
	Fajita, de cerdo o ternera	Fajita, beef or pork	58105050
	Tortitas, tipo *pancake*	Pancakes	18390
	Fideos orientales, Ramen	Instant noodles	6583
	Baba Ganush	Eggplant dip	75412030
	Vichyssoise	Vichyssoise	71801010
	Burrito	Burrito	21063
	Gyozas, vegetales	Gyoza, dumpling, with vegetables	071757010109

	Gyozas, con pollo	Gyoza, dumpling, chicken	74410975920
	Gyozas, con cerdo	Gyoza, dumpling, pork	78139710312
	Sushi roll, con salmón	Sushi roll, with salmon	58151200
	Sushi roll, vegetal	Sushi roll, with vegetables	58151230
	Poke Bowl	Poke Bowl	604580004504
	Croquetas, de jamón	Ham croquettes	75127003012
	Masa de hojaldre	Puff pastry	18211
	Masa de hojaldre, integral	Puff pastry, wholegrain, quiche	3528960013269
	Lasaña, vegetal	Lasagna, with vegetables	22956
	Canelones, vegetales	Cannelloni, with ricotta and spinach	810757011361
	Ratatouille	Ratatouille	2345555
	Empanada, de verdura	Pie, filled with vegetables	58116115
	Cebolla, frita	Crispy fried onions	688267078866
Pulses, seeds, and nuts	Edamame, cocinado	Edamame, cooked	11212
	Anacardo, frito	Cashew, salted	12586
	Crema de anacardo	Cashew butter, spread	12088
	Edamame, soja, tostado	Edamame, soy bean, roasted	64777815823
Sugar, chocolate, and related products	Cacao en polvo, 100%	Cocoa powder, unsweetened	19165
	Dulce de leche	Caramel, milk-based	1225
	Crema de Lotus	Lotus spread	21788506966
	Sacarina	Saccharin, sweetener	43158
	Stevia	Stevia, sweetener	19918

Supplemental Table 2. Overview of recipes and nutrient profiles for the 12 most commonly consumed dishes in Mallorca.

Recipe	Ingredients (g/person, raw)	Energy (kcal)/100 g	Fat (g)/ 100 g	Protein (g)/ 100 g	Carbs (g)/ 100 g	Fiber (g)/ 100 g
Vegetable puree	Pumpkin, 125 g<br>Leek, 70 g<br>Potato, 50 g<br>Onion, 62.5 g<br>Brie-type cheese, 12.5 g<br>Extra virgin olive oil, 5 ml<br>Salt<br>Water, 100 ml	65.09 kcal	3.23 g	2.14 g	6.80 g	2.29 g
Rice with toppings (*Rice "brut"*)	Rice, 60 g<br>Rabbit, 30 g<br>Chicken, 30 g<br>Pork, 60 g<br>*Butifarrón* sausage, 50 g<br>*Sobrasada* sausage, 10 g<br>Tomato, 80 g<br>Onion, 40 g<br>Green beans, 30 g<br>Garlic, 0.8 g<br>Virgin olive oil, 2 ml<br>Salt	155.53 kcal	8.05 g	7.51 g	13.15 g	0.59 g
Meatballs	Ground pork, 125 g<br>Onion, 50 g<br>Garlic, 0.5 g<br>Bread, 12.5 g<br>Wheat flour, 15 g<br>White wine, 31.2 g<br>Egg, 15 g<br>Virgin olive oil, 5 ml<br>Salt<br>Thyme<br>Parsley	240.54 kcal	16.75 g	12,48 g	9.99 g	0.96 g
Potato omelet	Chicken egg, 75 g<br>Potato, 150 g<br>Onion, 37.5<br>Virgin olive oil, 6.25 ml<br>Salt	126.26 kcal	6.53 g	5.75 g	11.05 g	1.41 g
Vegetables cake	Onion, 50 g<br>Peppers, 135 g<br>Wheat flour, 50 g	162.81 kcal	7.73 g	2.78 g	17.71 g	1.50 g

(*“Trampó” cake)*	Garlic 0.5 g<br>Extra virgin olive oil, 17.5 ml<br>Yeast<br>Salt<br>Water, 50 ml					
Mallorcan fried dish *(“Frito Mallorquin”)*	Lamb tongue, 20 g<br>Lamb heart, 20 g<br>Lamb *molleja*, 20 g<br>Lamb lung, 20 g<br>Lamb kidney, 20 g<br>Potato, 150 g<br>Red pepper, 66.7 g<br>Cauliflower, 50 g<br>Spring onion, 50 g<br>Garlic, 0.7 g<br>Fennel<br>Pepper<br>Extra virgin olive oil, 40 ml<br>Salt	192.04 kcal	16.12 g	6.71 g	8.77 g	1.67 g
Mallorcan soup (*“Sopas mallorquinas”*)	Tomatoes, 37.5<br>White cabbage, 250 g<br>Artichokes, 50 g<br>Mushrooms, 125 g<br>Bread 17.5 g<br>Virgin olive oil, 12.5<br>Salt	69.83 kcal	5.21 g	2.91 g	6.35 g	7.31 g
Seafood nodle (“Fideuà”)	Mussels, 25 g<br>Clams 32.5 g<br>Pasta, 60 g<br>Garlic clove, 10 g<br>White wine, 30 ml<br>Virgin olive oil, 12.5 g<br>Salt<br>Sweet paprika	150.98 kcal	7.42 g	6.43g	16.62 g	1.22 g
Quiche	Broccoli, 80 g<br>Egg, 30 g<br>Mushrooms, 40 g<br>Beets, 20 g<br>Milk, 25 ml<br>Spinach, 8 g<br>Mozzarella cheese, 20 g<br>Sheet of puff pastry, 46 g<br>Virgin olive oil, 10 ml<br>Garlic clove, 8 g	156.98 kcal	11.62 g	6.54 g	6.56 g	1.05 g

	Salt					
Risotto	Rice, 75 g<br>Mushrooms 100 g<br>Vegetable broth, 25 ml<br>Onion, 50 g<br>White wine 3 ml<br>Butter 7.5 g<br>Virgin olive oil, 12.5 g<br>Salt	149.81 kcal	5.99 g	3.06 g	20.5 g	0.8 g
Meat lentils	Lentils, 62.5 g<br>Chorizo sausage, 30 g<br>Tomato, 50 g<br>Onion, 50 g<br>Carrot, 50 g<br>Potato, 50 g<br>Garlic clove, 10 g<br>Extra virgin olive oil, 12.5 g<br>Salt	97.06 kcal	4.50 g	5.88 g	8.91 g	2.96 g
Vegetable lentils	Lentils, 62.5 g<br>Tomato, 50 g<br>Onion, 50 g<br>Garlic clove, 12.5 g<br>Carrot, 50 g<br>Potato, 50 g<br>Green beans, 50 g<br>Virgin olive oil, 12.5 g<br>Salt	81.83 kcal	3.46 g	4.68 g	9.90 g	3.46 g

Supplemental Table 3. Classification of Traqq-Spain food items based on the Spanish Food Frequency Questionnaire and WCRF/AICR recommendations

**Fruit and vegetables**	**Legumes**	**Wholegrains**	**Foods rich in fat, starch or sugars**	**Red meat**	**Processed meat**	**Sugar sweetened drinks**	**Alcoholic drinks**
Chard, garlic, apricot, artichokes, blueberry, etc.	Black beans, chickpeas, hummus, lentils, etc.	Brown rice, oat flakes, wholegrain bread, quinoa, etc.	Corn type appetizer, croquettes, industrial patty, sugary cereals, croissants, etc.	Pork chop, veal loin, minced meat, etc.	Bacon, turkey cold cuts, ham, salami, etc.	Soft drinks, juices, soluble cocoa, milkshakes, etc.	Wine, beer, liqueur, cider, etc.

Supplemental Figure 1. Distribution of completed 2hRs per participant

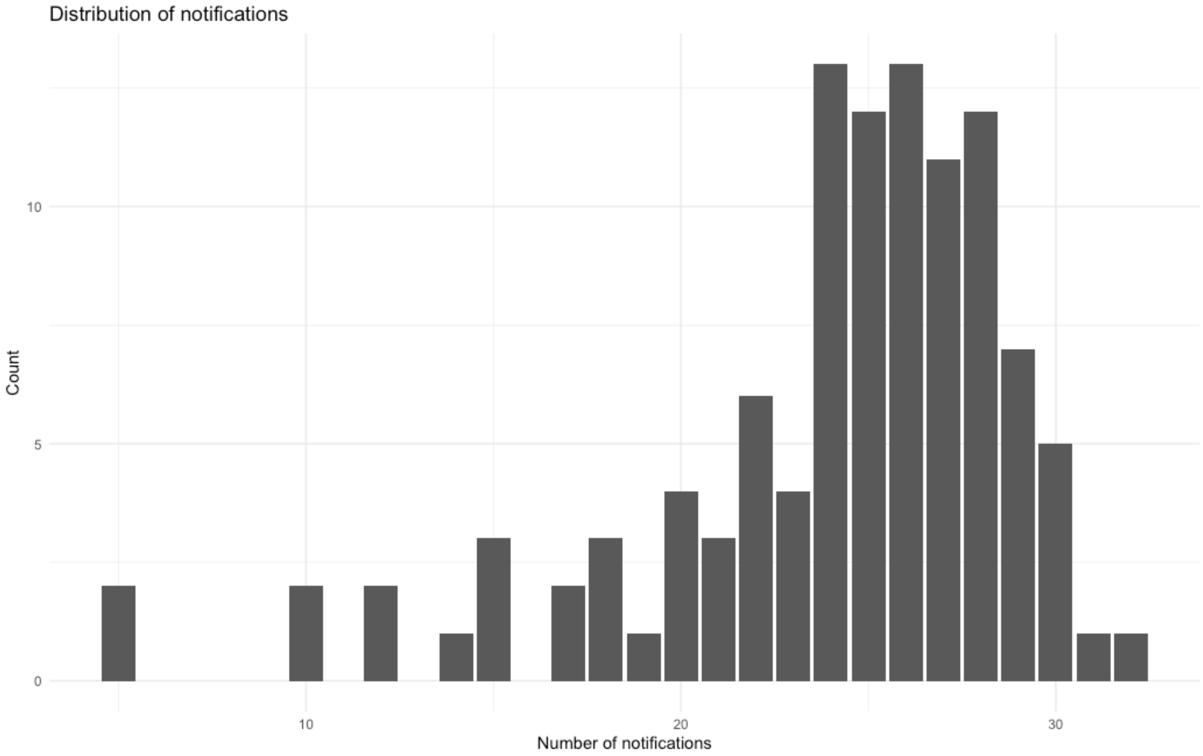

Supplemental Figure 2. Bland-Altman plot of the differences in intake for each the remaining food groups estimated with random 2hRs and FFQ, plotted against the mean of both methods. Mean difference (solid line), 95% limits of agreement (1.96 × SD of mean difference; dashed line), and linear regression line (blue dashed line) are included.

Figure C. Bland–Altman plot of agreement between 2hRs and FFQ for legumes (g/day)

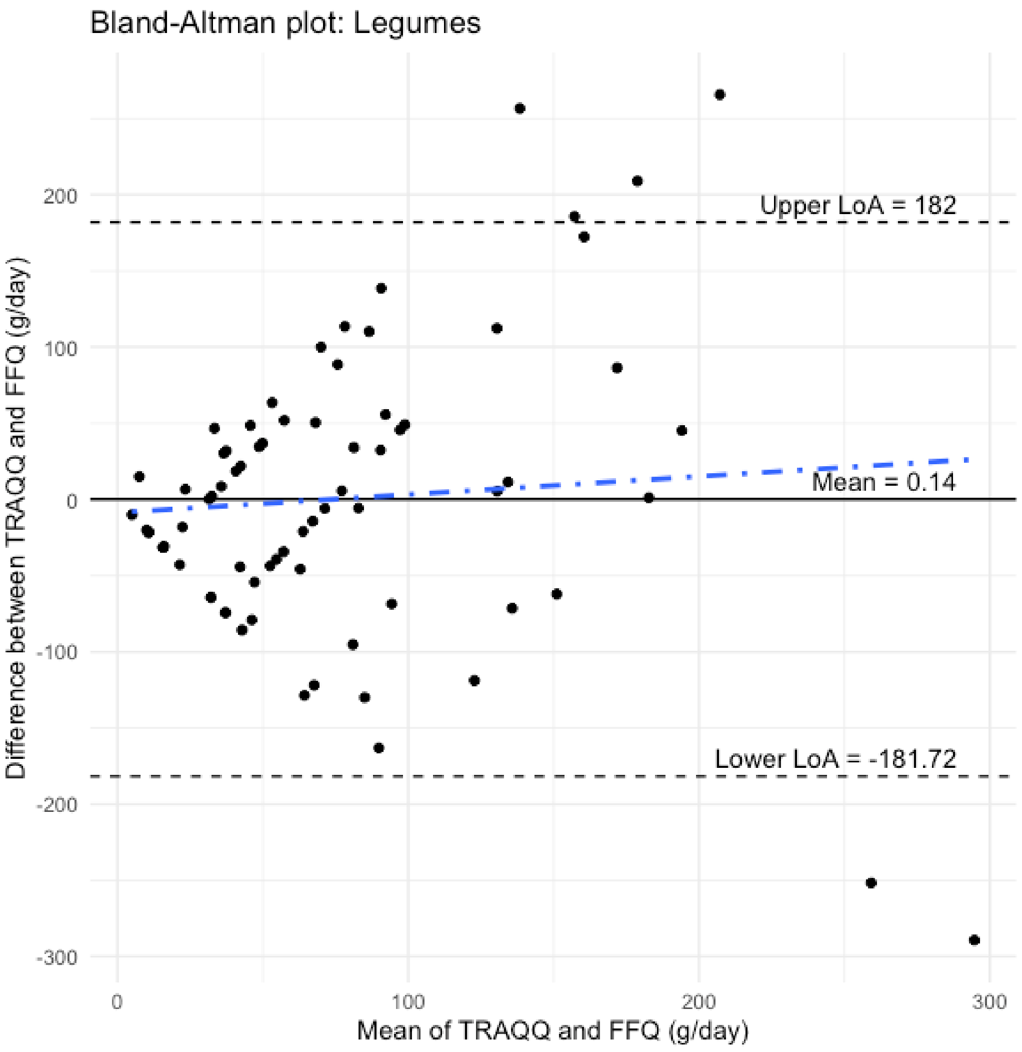

Figure D. Bland–Altman plot of agreement between 2hRs and FFQ for fast and processed foods (total g/day)

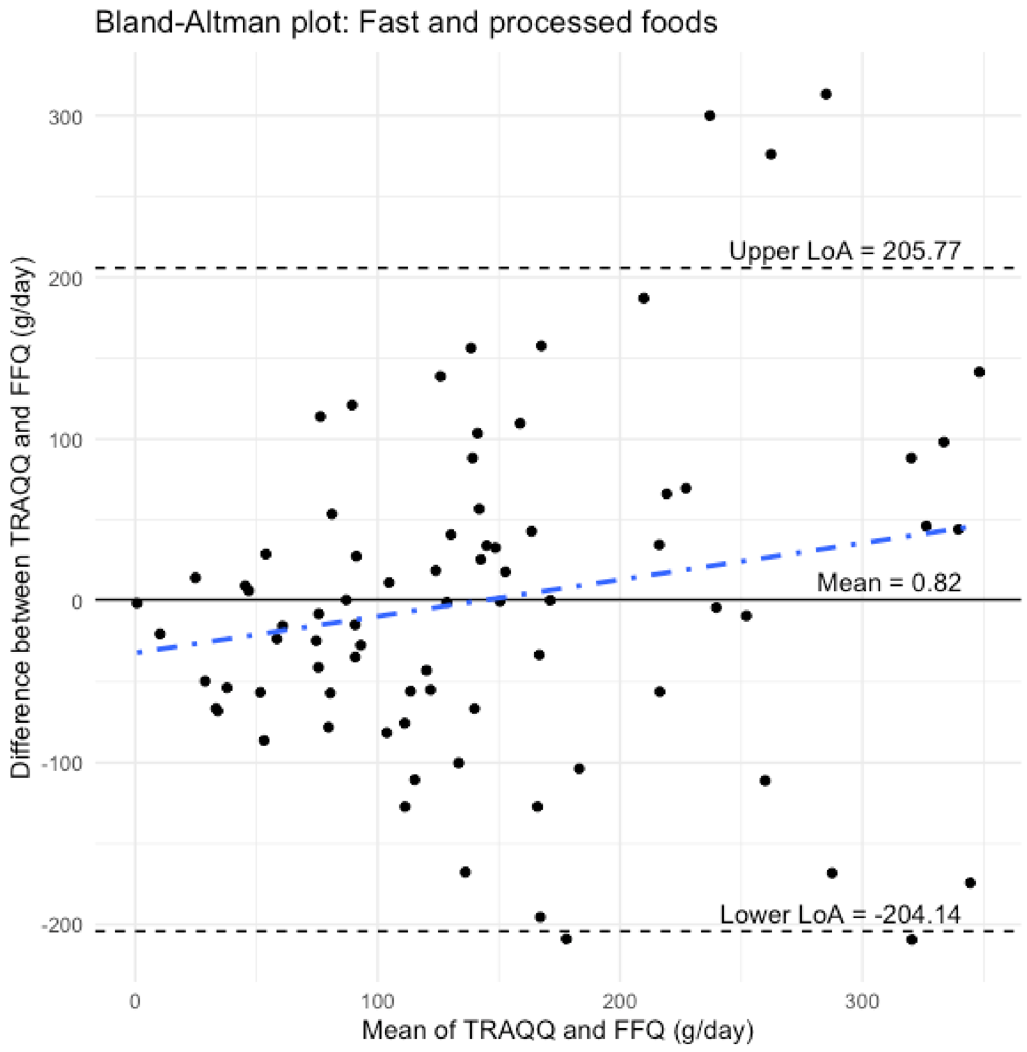

Figure E. Bland–Altman plot of agreement between 2hRs and FFQ for red meat (g/day)

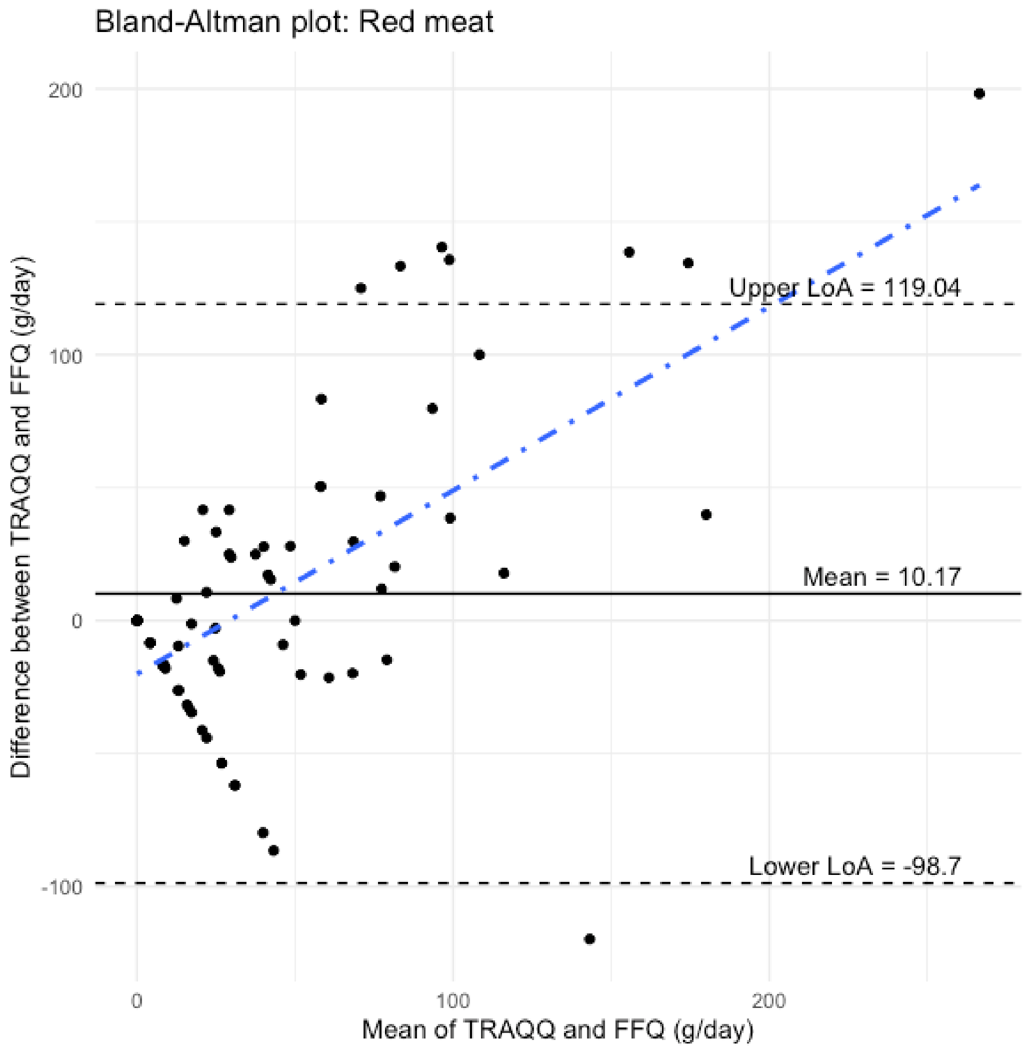

Figure F. Bland–Altman plot of agreement between 2hRs and FFQ for processed meat (g/day)

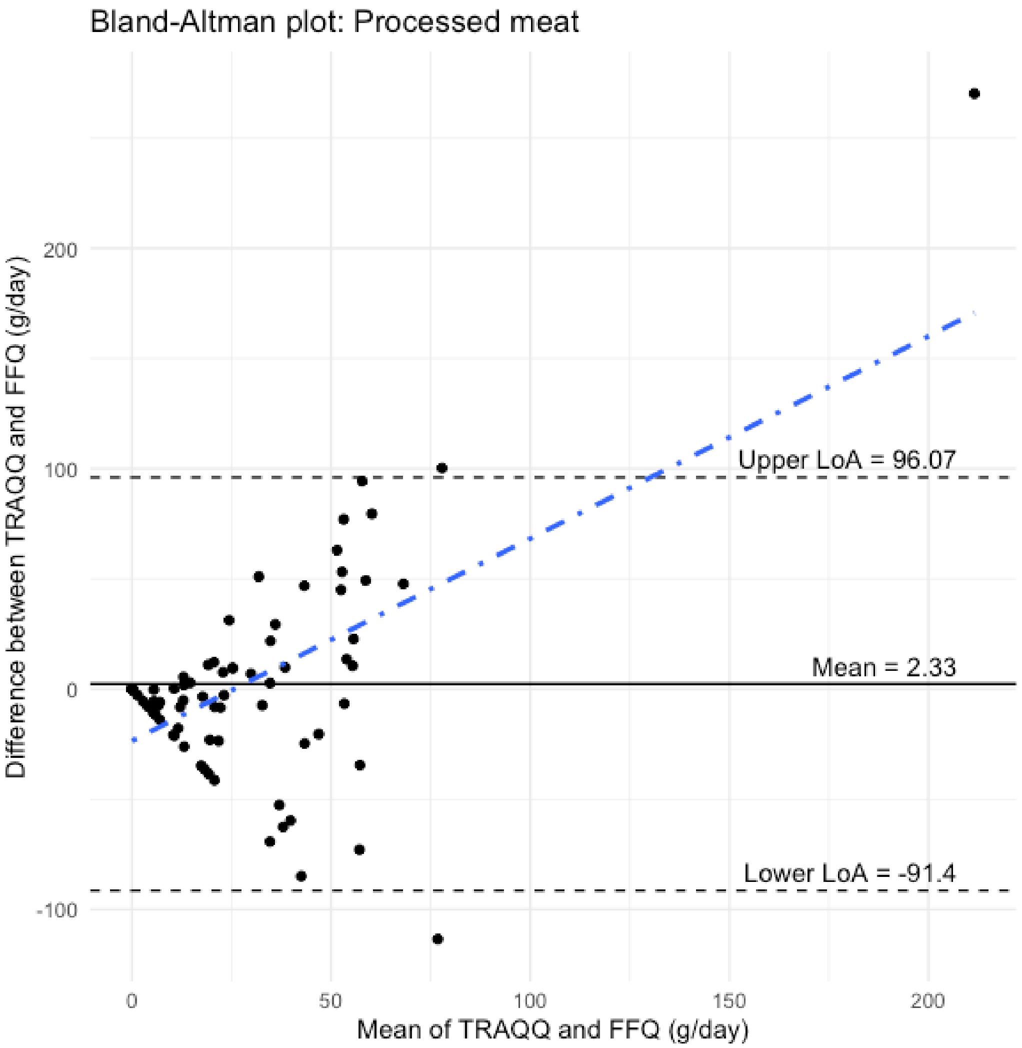

Figure G. Bland–Altman plot of agreement between 2hRs and FFQ for sugar-sweetened drinks (ml/day)

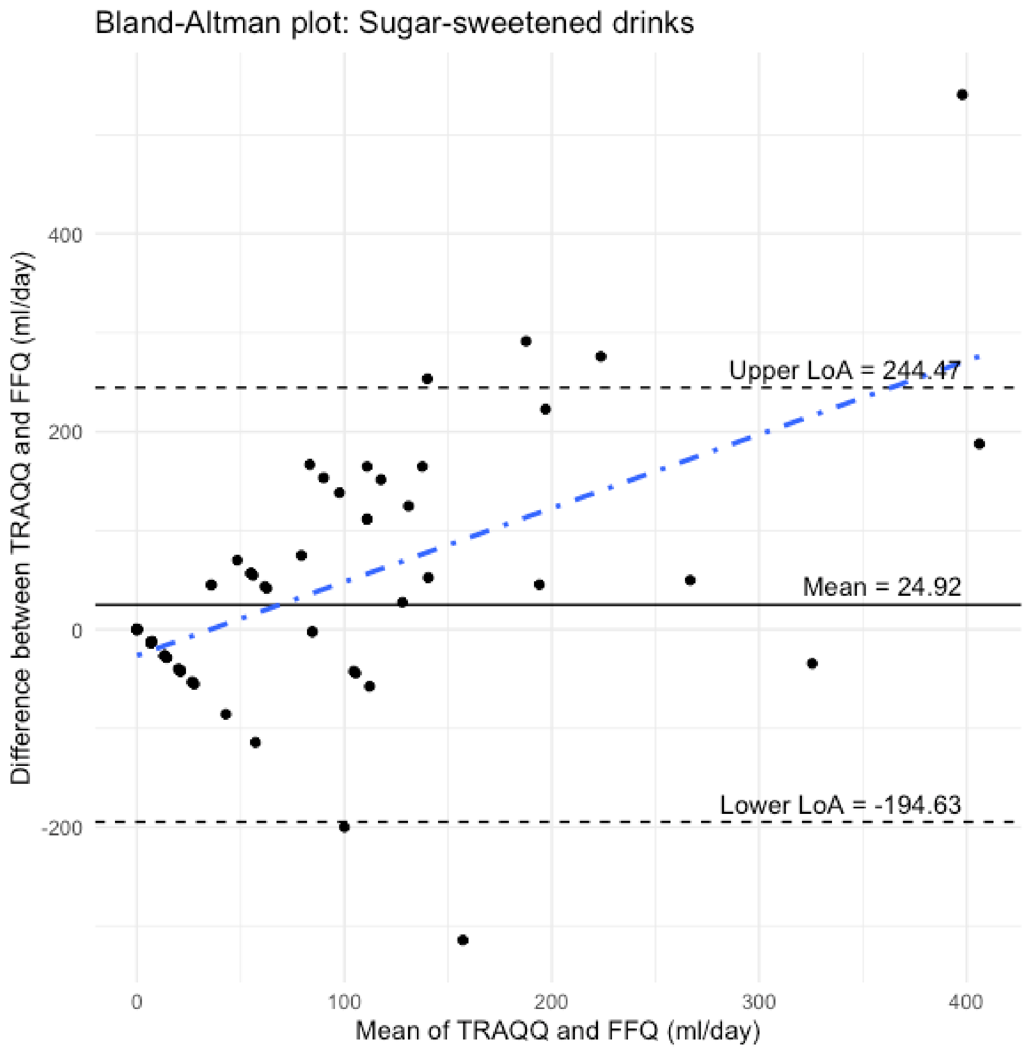

Figure H. Bland–Altman plot of agreement between 2hRs and FFQ for alcoholic drinks (ml/day)

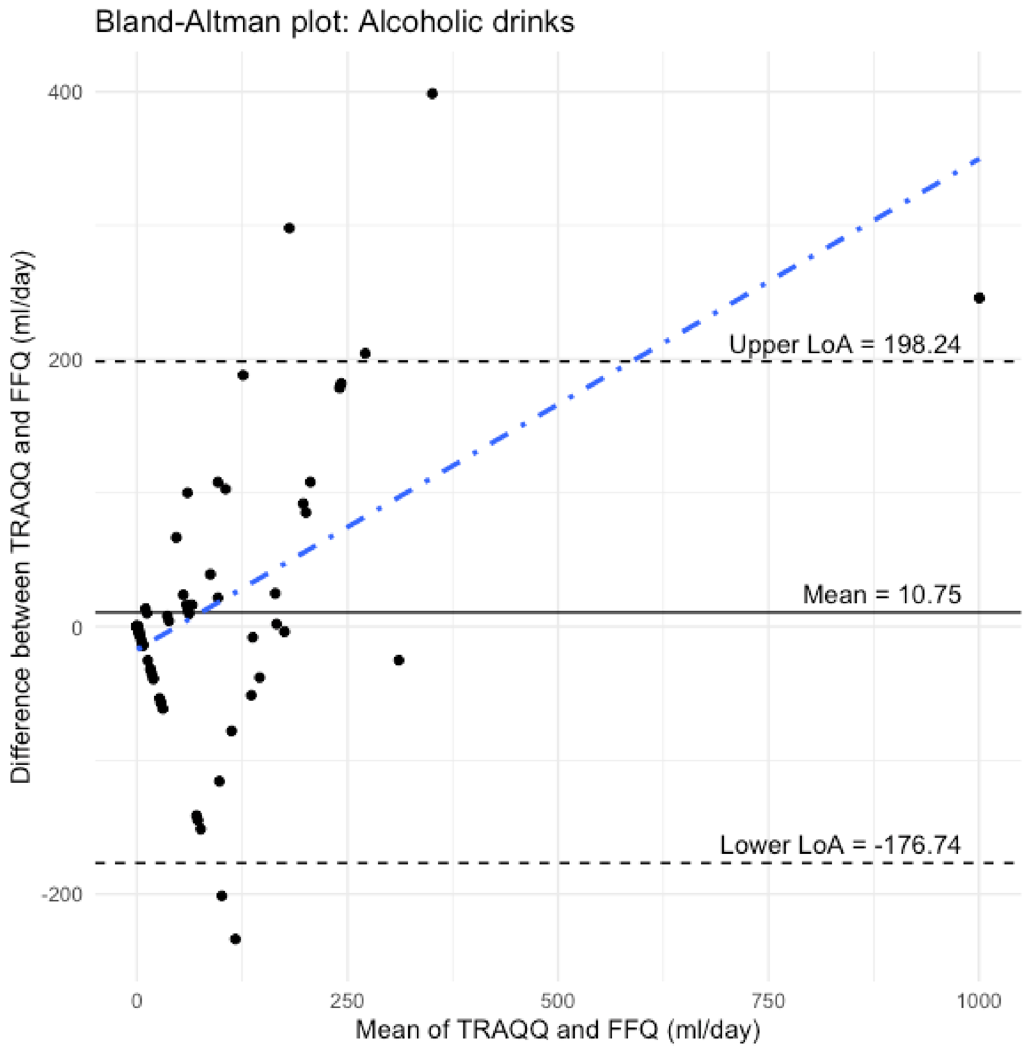

Supplement: Multimedia component 1 [file mmc1.pdf]
